# Sea Surface Temperature Prediction Enhanced by Exploring Spatiotemporal Correlation Based on LSTM and Gaussian Process

**DOI:** 10.3390/s25051373

**Published:** 2025-02-24

**Authors:** Zhenglin Li, Qingxiong Zhu, Dan Zhang, Hao Wu, Yan Peng

**Affiliations:** 1 School of Future Technology, Shanghai University, Shanghai 200444, China; zhenglin_li@shu.edu.cn (Z.L.); pengyan@shu.edu.cn (Y.P.); 2 Institute of Artificial Intelligence, Shanghai University, Shanghai 200444, China; 13028209416@shu.edu.cn; 3 School of Mechatronic Engineering and Automation, Shanghai University, Shanghai 200444, China; wuhao@shu.edu.cn

**Keywords:** sea surface temperature, probabilistic forecasting, spatiotemporal correlation, Gaussian Process Regression

## Abstract

The accurate prediction of sea surface temperature (SST) is essential for studying marine phenomena, understanding climate dynamics, and forecasting environmental changes. However, developing a general SST prediction model is challenging due to significant regional variations and the impacts of diverse climate phenomena. To improve the performance of SST predictions, we propose a hybrid framework that effectively models the spatial and temporal dependencies of SST data with a Gaussian process-enhanced Long Short-Term Memory network. The LSTM module adaptively captures both long and short-term temporal trends in SST variation, while the Gaussian process incorporates the spatial dependency of neighboring data to further refine the predictions. Furthermore, our proposed framework estimates the uncertainty associated with SST predictions, providing crucial information for practical applications. Comprehensive experiments are conducted on the OISST dataset, with a focus on the Bohai Sea and the South China Sea. The results of our framework outperform state-of-the-art methods, validating its superiority in SST prediction.

## 1. Introduction

Sea surface temperature (SST) is a vital parameter that characterizes the ocean’s thermal equilibrium, playing a crucial role in applications ranging from marine ecosystem management to global climate modeling [1,2]. With the rapid advancement of remote sensing technologies and in-situ observations, SST data have expanded significantly in both volume and complexity, introducing challenges for accurate prediction [3,4]. Therefore, leveraging advanced computational methods and big-data analytics becomes essential for managing and extracting valuable insights from these large-scale spatiotemporal datasets [5]. By processing these high-dimensional data, these methods contribute to more effective climate modeling and forecasting efforts [6,7]. Accurate SST prediction is therefore critical not only for scientific research but also for practical applications, such as mitigating the impacts of climate variability and improving forecasts of phenomena like the El Niño Southern Oscillation [8,9,10].

Methods for predicting SST can be broadly categorized into two types: physical dynamic models and data-driven approaches. Currently, there are over 40 marine numerical models, each designed with specific advantages and tailored for distinct applications, as highlighted in recent studies [11]. Prominent models for SST prediction include the General Circulation Model (GCM) [12], the Integrated Forecast System (IFS) [13], and the Global Forecast Systems (GFS) [14]. The GCM, for instance, simulates global climate changes by calculating atmospheric evolution based on conservation laws. In contrast, the IFS and GFS, developed by the European Centre for Medium-Range Weather Forecasts (ECMWF) and the National Centers for Environmental Prediction (NCEP) respectively, offer SST predictions across varying time scales. Additionally, the Hybrid Coordinate Ocean Model (HYCOM) [15,16] incorporates three types of self-adaptive coordinates, enhancing its versatility. Despite these advancements, the precision of high-resolution SST predictions by physical dynamic models remains constrained by imperfect parameterization schemes, the limitations of observational data, and the inherent complexity of oceanic processes.

Leveraging big data, data-driven approaches offer a powerful means of autonomously uncovering patterns within vast SST datasets, reducing the need for detailed domain expertise [17,18]. These methods include traditional machine learning models like vector autoregressive models [19], autoregressive integrated moving average (ARIMA) [20], and support vector machines (SVMs) [21]. For instance, Lavine [22] employed Markov random fields for ocean temperature analysis, while Aguilar-Martinez [23] utilized support vector regression (SVR) for predicting monthly SST anomalies. However, these models often encounter difficulties with long-term trend prediction, and their accuracy diminishes as the forecasting horizon extends [24].

The recent proliferation of observational data from in-situ measurements and satellite remote sensing has driven significant advancements in deep neural networks for SST prediction [25,26,27,28,29]. Recurrent neural networks (RNNs), particularly Long Short-Term Memory (LSTM) networks, have been successfully applied to capture the intricate temporal dynamics of SST [30,31]. Zhang et al. [32] proposed the FC-LSTM model, which integrates LSTM with fully connected layers for single-point SST prediction. Yang et al. enhanced the FC-LSTM model by incorporating convolutional neural networks to predict SST, considering spatial correlation and utilizing fixed-dimensional patches to capture the local correlation and global coherence of each pixel [33]. Usharani introduced a novel loss function for the LSTM-based SST prediction model, aiming to minimize errors and expedite convergence [34]. Xiao et al. introduced the LSTM-Adaboost method that combines the deep recurrent-neural-network model of LSTM with the AdaBoost ensemble learning model to address potential overfitting issues in LSTM and improve accuracy in short- and medium-term daily SST prediction [35]. Han et al. adopted ConvLSTM [36] for end-to-end SST prediction training, substituting the matrix multiplication in FC-LSTM with convolutional operations [37]. Additionally, Araújo et al. [38] introduced a novel deep neural-network architecture leveraging Dilation–Erosion–Linear (DEL) processing units for forecasting sea surface temperatures (SST). This model, grounded in the integration of linear and nonlinear components through mathematical morphology and gradient-based learning, aims to enhance SST prediction by capturing long-term dependencies and nonlinear patterns within the time-series data.

These SST prediction methods mainly focus on capturing temporal patterns, but hardly consider the spatial correlation between individual SST values or their insufficient ability to extract spatial information [39]. In specific regions, like landmasses or islands, the scarcity of reliable SST data complicates the accurate encoding of spatial SST variability via convolutional neural networks. SST prediction methods that integrate spatiotemporal information can address performance bottlenecks inherent in traditional approaches. Moreover, offering reliable uncertainty estimates through probabilistic assessments is highly advantageous [40]. Recently, several methods have emerged that employ graph neural networks to capture SST’s spatial features, as seen in [41,42,43,44]. The application of graph neural networks in predicting sea surface temperature (SST) has demonstrated promising results, owing to its ability to capture spatial features, adaptability across diverse regions, and integration of spatiotemporal information [45]. However, these approaches demand significant data and computational resources and often fall short in offering uncertainty metrics for their predictions.

In this paper, we propose a hybrid LSTM–Gaussian Process Regression (LSTM-GPR) framework for SST prediction, leveraging the spatiotemporal correlation of historical and neighboring SST data. The proposed model comprises two main modules: the temporal dependency extraction module and the spatial dependency enhancement module. The former discerns inherent temporal patterns in the SST data through an LSTM network, by extracting informative historical features and generating accurate predictions for each data-sampling location. The latter module refines the predicted SST of the target point by establishing a probabilistic distribution to model the dependence between the temperature of the center point and its spatially adjacent data.

The key contributions of this paper can be summarized as follows:

(1) The proposed LSTM-GPR framework effectively models the spatiotemporal patterns of SST, enabling accurate predictions. The Gaussian process models spatial correlations based on predicted SST values and their surrounding areas, while the LSTM captures temporal dynamics.
(2) Our model provides valuable information on prediction uncertainties by leveraging the inherent advantages of Gaussian processes to estimate confidence intervals for SST forecasting.
(3) The results of extensive experiments on the OISST dataset validate the superiority of our framework over existing methods.

The rest of the paper is arranged as follows. Section 2 presents the proposed LSTM-GPR framework in detail. Next, the experimental results are illustrated and analyzed in Section 3. Finally, Section 4 concludes the paper.

## 2. Methodology

### 2.1. Problem Formulation

**Figure 1:**
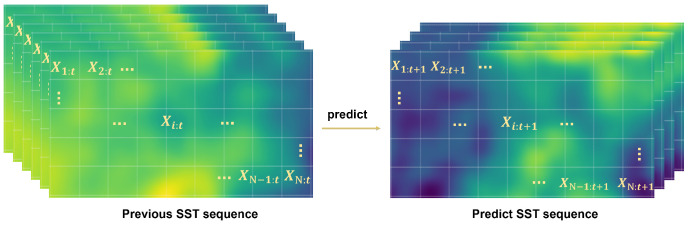
Problem formulation.

SST forecasting leverages historical temperature records from in-situ observations and reanalysis, complemented by other oceanographic variables, to predict upcoming temperature fluctuations. Oceanic data are organized into grids based on longitude and latitude coordinates, with each grid unit signifying a unique observation point. Consider a temporal series of ocean data for a region denoted as $X=\{X_{:,0},X_{:,1},\cdots ,X_{:,t}\}$, where $X_{:,t}=\left\{X_{1,t},X_{2,t},\cdots ,X_{N,t}\right\}\in R^{N\times d}$ encapsulates the data from *N* points at time *t* within the region, with *d* representing the data dimension. The problem formulation is depicted in Figure 1. For the SST prediction, we use the preceding data of the *k* steps of time *t* in the sequence *X* as historical window. These data are used to forecast the SST value for the subsequent τ time steps after *t*, as follows:

(1) $$X_{:,t+1},\cdots ,X_{:,t+\tau }=F\left(X_{:,t-k+1},X_{:,t-k+2},\cdots ,X_{:,t};\theta \right),$$

where θ represents all the learnable parameters in the prediction model *F*.

In addition to the primary SST predictions, it is imperative to quantify the confidence associated with these forecasts. This confidence measure serves as an indicator of the reliability and robustness of the predictions, allowing users to make informed decisions based on the model’s outputs. Specifically, for each predicted value, a confidence interval (CI) is computed. The CI provides a range within which the true value is likely to fall with a specified probability. In this study, we focus on the 95% confidence interval, meaning there is a 95% chance that the true value lies within this interval.

Mathematically, for a predicted value $\hat{y_{i}}$ at time step *t*, the 95% confidence interval is given by:

(2) $$\hat{y_{i}}\pm z\times \mathrm{SE}$$

where *z* is the z-score corresponding to the desired confidence level (1.96 for 95% CI) and SE is the standard error of the prediction. The standard error can be derived from the model’s residuals or, in the case of probabilistic models, directly from the model’s output.

Furthermore, the probability density function (PDF) associated with each prediction offers a visual representation of the likelihood of different outcomes, further elucidating the prediction’s uncertainty. Incorporating these confidence measures not only enhances the credibility of the predictions but also provides a comprehensive understanding of the model’s performance and its potential limitations.

### 2.2. Overview of the LSTM-GPR SST Prediction Framework

**Figure 2:**
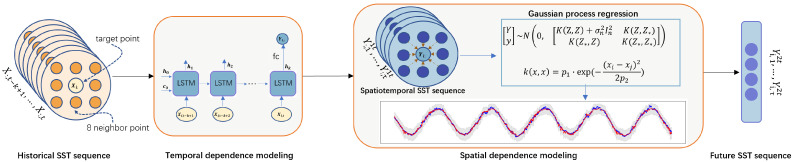
Workflow of LSTM-GPR.

Oceanic points exhibit intricate temporal and spatial correlations due to their inherent inter connectivity. Accurately capturing these correlations is pivotal for enhancing the precision of oceanic temperature predictions at specific locations. Moreover, in practical applications, it is not just the prediction that matters but also the confidence with which these predictions are made. To address these challenges, we introduce the LSTM-GPR framework, a hybrid model that synergistically combines the temporal prediction strengths of Long Short-Term Memory (LSTM) networks with the probabilistic forecasting capabilities of Gaussian Process Regression (GPR). A schematic of the LSTM-GPR SST prediction framework is depicted in Figure 2.

The proposed framework operates in two distinct phases: temporal dependency extraction and spatial dependency enhancement.

In the initial phase, historical SST sequences are input into the model. This module, equipped with LSTM layers and fully connected layers, is trained to discern and capture inherent temporal patterns within the data. The objective is to extract meaningful spatiotemporal features from the SST sequences, emphasizing the temporal dimension.

In the subsequent phase, once temporal features are extracted, the focus shifts to the spatial dimension. Here, the Gaussian process plays a pivotal role. It considers the spatial relationships among SST points, refining the accuracy of the predictions. Notably, this phase does not just produce point estimates but also generates confidence intervals for the predictions, offering insights into the reliability of the forecasted values.

A unique strength of our approach is its adaptability to diverse geographical contexts, including coastal regions. For coastal points, where data might be sparse or irregular, our model effectively simulates the spatial information of a central point by leveraging data from its surrounding valid points. This ensures that the model remains robust and efficient, even in challenging terrains like coastlines.

### 2.3. Temporal Dependency Extractor and LSTM Mechanism

The temporal dependency extractor is designed to capture both the trend and seasonal patterns (i.e., long-term dependencies), as well as the non-stationary features (i.e., short-term fluctuations) in the SST sequences. Specifically, we employ Long Short-Term Memory (LSTM), an advanced form of recurrent neural network (RNN) that integrates non-linear and data-dependent control mechanisms. This design helps overcome the common challenge of vanishing gradients in deep neural-network training, especially for longer sequences [46].

**Figure 3:**
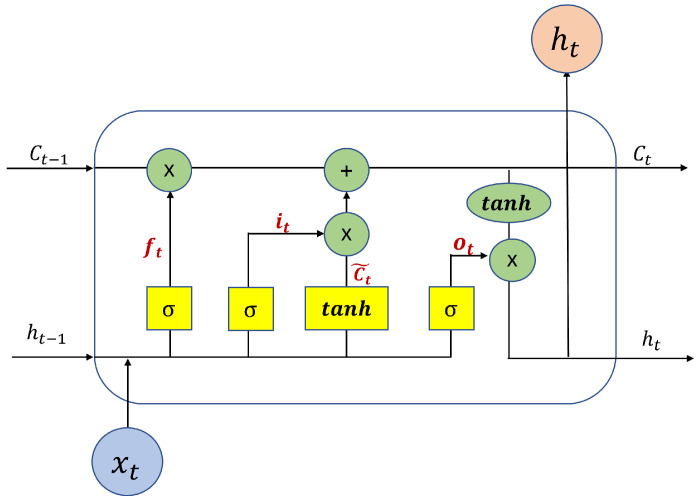
LSTM cell structure.

The dual state mechanism of LSTM cells facilitates the modeling of complex temporal patterns in SST sequential data. The *c* state captures long-term dependencies, reflecting broader trends and seasonal patterns, while the hidden state *h* addresses short-term fluctuations and anomalies. This unique structure of LSTM allows for the maintenance and updating of its state over time, providing a powerful tool for time-series analysis where the continuity of context and the significance of recent events are crucial. The architecture of an LSTM cell is illustrated in Figure 3.

LSTM employs three principal gates: the forget gate ($f_{t}$), input gate ($i_{t}$), and output gate ($o_{t}$), as depicted in Figure 3. The forget gate decides the amount of past information to retain, the input gate determines which new information should be stored, and the output gate controls the amount of information passed from the cell state to the hidden state. The mathematical operations for these gates are as follows:

(3) $$\begin{array}{cc} f_{t} & =\sigma (W_{fx}x_{t}+W_{fh}h_{t-1}+b_{f})\\ i_{t} & =\sigma (W_{ix}x_{t}+W_{ih}h_{t-1}+b_{i})\\ {\tilde{c}}_{t} & =\tanh(W_{cx}x_{t}+W_{ch}h_{t-1}+b_{c})\\ c_{t} & =f_{t}⊙c_{t-1}+i_{t}⊙{\tilde{c}}_{t}\\ o_{t} & =\sigma (W_{ox}x_{t}+W_{oh}h_{t-1}+b_{o})\\ h_{t} & =o_{t}⊙tanh\left(c_{t}\right) \end{array}$$

where *W* and *b* are the trainable parameters for each gate, and ⊙ denotes element-wise multiplication.

After processing through the LSTM, the state vector $h_{t}$ is passed to a fully connected layer to predict the SST value for the upcoming τ days. The LSTM operations can be succinctly represented as:

(4) $$h_{t},c_{t}=\mathrm{LSTM}\left(h_{t-1},c_{t-1},X_{i,t},W\right)$$

(5) $$Y_{i,:}^{1t}=\sigma \left(W_{fc}h_{l}+b_{fc}\right)$$

where $X_{i,:}$ represents the previous SST sequence of the *i*th point, $Y_{i,:}^{1t}\in R^{\tau }$ is the predicted value of future τ days at time *t*. This methodology facilitates the generation of a future τ-day spatiotemporal SST sequence for the entire region.

### 2.4. Spatial Dependency Enhancement with GPR

The spatial autocorrelation between geographically proximate sampling points is influenced by factors like air flow, ocean currents, and water temperature. This results in spatial continuity and similarity. To harness this spatial dependency for improving SST predictions, we employ Gaussian Process Regression (GPR) in the second stage of our framework.

GPR models the relationship between input and output variables using a Gaussian process, which defines a prior distribution over functions. Once observations are available, this model is updated, leading to the derivation of a posterior distribution. By selecting spatially proximate sample points as input features, the GPR model can better capture the spatial dependencies within a region, thereby enhancing prediction accuracy. This is especially beneficial for coastal points, where the model can improve forecast accuracy by using data from surrounding valid points and excluding land data.

Specifically, the training set $D={\left\{\left(z_{t},y_{t}\right)\right\}}_{t=1}^{T}=\left(Z,Y\right)$ is assumed, where $z_{t}\in R^{d}$ represents the input feature vector comprising the target point $Y_{i,\tau }^{1t}$ at time *t* from the spatiotemporal SST sequence and its adjacent valid points $Y_{:,\tau }^{1t}$, and $y_{t}\in R$ denotes the actual measurement value of the future τ day of the target point at time *t*. We assume the prediction model is as follows:

(6) Y=f(Z)+ε

where f(·) is a Gaussian process characterized by its mean and covariance function, and $\varepsilon ∼N(0,\sigma^{2})$ is a noise term. The prior distribution of *Y* can be represented as follows, where the mean function is typically set to 0.

(7) $$Y∼N(0,K\left(Z,Z\right)+\sigma_{n}^{2}I_{n})$$

The covariance matrix $K\left(z,z\right)=\left(\kappa_{ij}\right)$ is a symmetric positive definite matrix, where each element $\kappa_{ij}$ is generated by a kernel function that quantifies the correlation between $z_{i}$ and $z_{j}$ for every pair of data samples. $I_{n}$ is an n-dimensional identity matrix. Considering $\left(Z_{*},Y_{*}\right)$ as the testing set and its corresponding predicted value, we can derive the joint prior distribution of the observed values *Y* and the predicted value $Y_{*}$.

(8) $$\left[\begin{array}{c} Y\\ Y_{*} \end{array}\right]∼N\left(\begin{array}{c} 0,\left[\begin{array}{c} K\left(Z,Z\right)+\sigma_{n}^{2}I_{n}\;K\left(Z,Z_{*}\right)\\ K\left(Z_{*},Z\right)\;K\left(Z_{*},Z_{*}\right) \end{array}\right] \end{array}\right)=N\left(\begin{array}{c} 0,\left[\begin{array}{c} K\;K_{*}^{T}\\ K_{*}\;K_{**} \end{array}\right] \end{array}\right)$$

$K\left(Z_{*},Z\right)=K{\left(Z,Z_{*}\right)}^{T}$ is the covariance matrix between the test set $Z_{*}$ and training set *Z*. $K(Z_{*},Z_{*})$ is the covariance matrix of the test set itself. Squared exponential kernel, linear kernel, and polynomial kernel are all common kernel functions. The formula of squared exponential kernel is as follows. $p_{1}$ and $p_{2}$ are hyperparameters.

(9) $$\kappa_{ij}=p_{1}\cdot exp\left(-\frac{{\left(z_{i}-z_{j}\right)}^{2}}{2p_{2}}\right)$$

The posterior distribution can be derived by leveraging the conditional distribution property of Gaussian distribution.

(10) $$Y_{*}|Y∼N\left(\bar{Y},\sigma^{2}\right)$$

(11) $$\bar{Y}=K_{*}K^{-1}Y$$

(12) $$\sigma_{y}^{2}=K_{**}-K_{*}K^{-1}K_{*}^{T}$$

$Y_{i,t}^{2t}\in R^{\tau }$ is refined by its spatial correlation of adjacent points $Y_{:,t}^{1t}\in R^{\tau }$ from each step’s prediction results based on Gaussian process.

Beyond point predictions, our model’s capability to provide interval and probabilistic forecasts stands out as a significant advantage. In real-world applications, understanding the uncertainty associated with predictions can be as crucial as the predictions themselves. For instance, when considering marine activities or planning interventions based on SST forecasts, knowing the range within which temperatures might fluctuate can aid in making informed decisions. Our GPR-based approach provides a 95% confidence interval for predictions, offering a range within which the actual values are likely to fall. This interval prediction is particularly useful for risk assessment and management. Furthermore, the model’s ability to generate a probability density function (PDF) for each predicted value offers insights into the likelihood of specific outcomes, allowing stakeholders to gauge the most probable scenarios and plan accordingly. The PDF formula is as follows:

(13) $$p\left(Y_{i}\right)=\frac{1}{\sqrt{2\pi }\sigma_{Yi}}\exp\left(-\frac{\left(Y_{i}-\bar{Y_{i}}\right)}{2\sigma_{Yi}^{2}}\right)$$

By effectively harnessing the spatial autocorrelation inherent in oceanic data, our model achieves enhanced accuracy, especially in challenging regions like coastlines. Coupled with its ability to quantify uncertainties and provide probabilistic forecasts, the LSTM-GPR framework emerges as a comprehensive solution for SST prediction, catering to both scientific inquiries and practical applications.

## 3. Experiments

### 3.1. Datasets

In our endeavor to demonstrate the capabilities of the LSTM-GPR model, we focus on capturing the intricate spatiotemporal correlations present in climate variables. The Bohai Sea and the South China Sea serve as the primary regions for our case studies, offering a diverse and challenging environment to validate the efficacy of our model. For our experiments in the Bohai Sea and South China Sea regions, we selected SST, USSW, and VSSW as the predictor variables for SST.

Our primary SST data are sourced from the National Oceanic and Atmospheric Administration (NOAA) OISST dataset [47]. This comprehensive dataset provides daily, weekly, and monthly average grid data of sea surface temperatures, offers global coverage of the oceans. The granularity of the data is commendable, with a spatial resolution of 0.25° × 0.25°, ensuring detailed insights into sea surface temperatures.

To complement our SST data, we incorporated wind-speed metrics (measured in m/s), specifically USSW (east–west wind speed) and VSSW (north–south wind speed). These metrics were extracted from the CCMP V02.0 dataset, providing a holistic view of the atmospheric conditions influencing sea temperatures. We curated sub-datasets from the OISST and CCMP datasets, focusing exclusively on the Bohai Sea and South China Sea regions. These curated data span a substantial period, covering three decades from 1990 to 2019, which translates to a total of 10,592 days of data. The initial 80% of the dataset, constituting the bulk of our historical records, was earmarked for training. The subsequent 20%, which offers more recent data, was set aside for testing, ensuring that our model’s predictions are evaluated against the most contemporary and relevant data.

### 3.2. Experiment Setup

To validate the efficacy of the proposed LSTM-GPR model, we benchmarked its performance against established methods: SVR [23], GPR, and LSTM. The OISST was employed to forecast daily mean sea surface temperatures for a horizon of 1–7 days. Importantly, the dataset was chronologically ordered, ensuring that the temporal relationships within the data were preserved. The model was designed to take 10 days of SST as input and produce forecasts for the subsequent 7 days. The choice of a 10-day sliding time window was made because it aligns with the window length used in similar studies, and it provides a reasonable balance between capturing temporal trends and minimizing data complexity. For the 1–7 day forecast horizon, a 10-day window effectively captures both short-term and longer-term trends without introducing unnecessary complexity, and longer windows did not significantly improve performance.

The model’s predictive performance was quantified using three widely-accepted metrics: Root Mean Square Error (RMSE), Mean Absolute Error (MAE), and the Coefficient of Determination ($R^{2}$). These metrics offer a comprehensive assessment, capturing both the magnitude and directionality of prediction errors. All of the test results below are from the test set, which is the last 20% of the total time series. The respective formulas for these metrics are:

(14) $$\mathrm{RMSE}=\sqrt{\frac{1}{n}\sum_{i=1}^{n}{\left(y_{i}-\hat{y_{i}}\right)}^{2}}$$

(15) $$\mathrm{MAE}=\frac{1}{n}\sum_{i=1}^{n}\left|y_{i}-\hat{y_{i}}\right|$$

(16) $$R^{2}=1-\frac{\sum_{i=1}^{n}{\left(y_{i}-\hat{y_{i}}\right)}^{2}}{\sum_{i=1}^{n}{\left(y_{i}-\bar{y}\right)}^{2}}$$

where $y_{i}$ denotes the observed value $\hat{y_{i}}$ represents the predicted result of the model, and *n* is the total number of test-set samples. Lower RMSE and MAE values signify superior model stability and accuracy, while a higher $R^{2}$ value indicates better predictive performance.

Prior to feeding the data into the LSTM model, they underwent a normalization process to ensure consistent scale and distribution. This involved centering the data around their mean and scaling based on their standard deviation. The normalization formula is:

(17) $$x_{\mathrm{norm}}=\frac{x-\mu }{\sigma }$$

It is crucial to note that the normalization parameters (mean μ and standard deviation σ) were computed exclusively from the training set to prevent data leakage. The normalized data then served as the input for the LSTM model.

### 3.3. Experiment Results and Discussion

#### 3.3.1. Evaluation of the Model Performance

**Figure 4:**
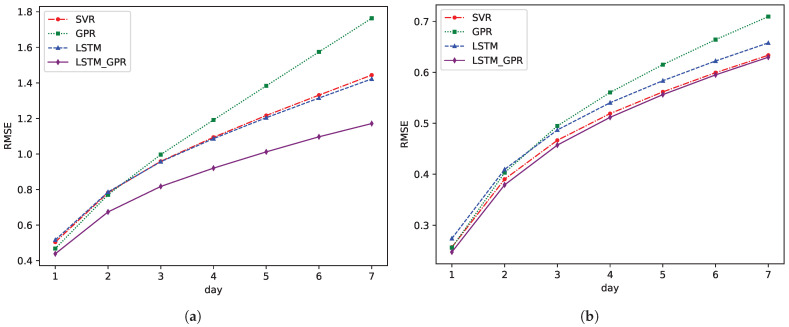
RMSE of different methods for 7-day sea surface temperature prediction. (**a**) Bohai Sea. (**b**) South China Sea.

To assess the performance of our proposed model, we began by employing a single-step rolling prediction for SST using one variable across four distinct methods. In this experimental setup, the actual SST values from the preceding 10 time steps were consistently utilized as historical inputs for the various models. These models then predicted the SST values for the subsequent time step. After each prediction, the predicted value was added to the historical dataset, and the model then used the most recent 10 SST values to forecast the next step. The results of this rolling prediction are illustrated in Figure 4, which displays the RMSE values for the four methods over a seven-day period in both the Bohai Sea and the South China Sea regions.

A salient observation from the results is the distinct challenge posed by the Bohai Sea region. Its multifaceted coastline, coupled with diverse climatic conditions, introduces substantial temperature variations, making SST predictions particularly challenging. The Bohai Sea’s shallow depth, semi-enclosed nature, and significant river discharge, especially from the Yellow River, create considerable seasonal and spatial variability in SST. These factors contribute to the increased complexity of accurately forecasting SST in the Bohai Sea. In contrast, the South China Sea, being closer to the equator and having a deeper, more expansive area, experiences relatively more stable climatic conditions and less seasonal variability. This results in a more uniform RMSE distribution across methods and greater predictability of SST values in the region.

From our results, it is evident that relying solely on Gaussian process prediction struggles to accurately capture the intricate patterns of SST changes. A standalone Gaussian process, although robust in many applications, seemed to grapple with the complexities of SST patterns, especially in regions with pronounced climatic variations. However, when we amalgamated the Gaussian process’s spatial-feature-extraction capabilities with the LSTM’s temporal prowess, the results were markedly improved. As shown in Figure 4, the LSTM-GPR model not only consistently outperformed other methods but also demonstrated robustness in handling the increased uncertainty associated with longer forecast horizons. This superiority was especially pronounced in the Bohai Sea region, where other prediction models demonstrated noticeable limitations.

To provide a more precise comparison of their prediction accuracy, Table 1 showcases the prediction accuracy metrics for a forecast horizon of 1–7 days across both the Bohai Sea and South China Sea regions using the four methods. Entries in bold highlight the superior performance among the methods. A discernible observation from the table is the consistent supremacy of our LSTM-GPR model over its counterparts in both datasets.

#### 3.3.2. Visualization of Prediction Results

**Figure 5:**
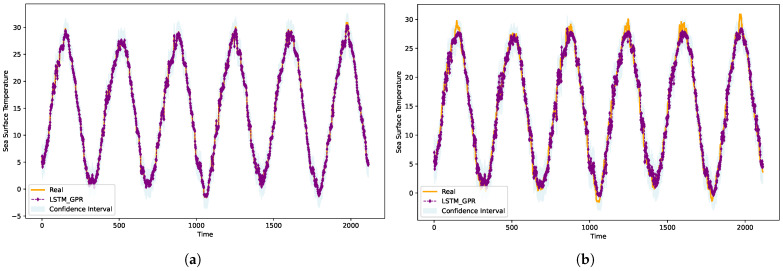
Comparison of true and estimated SSTs for 1-day (**a**) and 7-day (**b**) forecasts using the LSTM-GPR model.

Figure 5a,b provides a visual representation of the prediction outcomes of our LSTM-GPR model for both day 1 and day 7 within randomly selected locations in the Bohai Sea region. To enhance the interpretability of the model’s predictions, we have incorporated a 95% confidence interval in the visualization. The depicted purple line traces the model’s predictions, while the solid orange line delineates the actual observed values.

A salient observation from the visualizations is the pronounced cyclical fluctuation of SST in the Bohai Sea, with temperatures oscillating between 0 to 30 degrees across different years. A closer inspection reveals that the predictions in Figure 5a, representing a one-day forecast, align more closely with the actual SST values compared to those in Figure 5b, which showcases a seven-day forecast horizon. This longer forecast horizon naturally introduces more challenges due to the increased unpredictability of marine processes and seasonal variations, which can lead to larger deviations. However, our model consistently demonstrates resilience in maintaining high accuracy even as forecast horizons extend.

A noteworthy pattern emerges during the summer months, where the prediction errors tend to amplify. This can be attributed to the heightened sea temperatures during this period, which induce rapid, less predictable processes and potentially extreme weather events. In contrast, the winter months, characterized by colder sea temperatures, lead to more gradual processes, resulting in more accurate and consistent predictions. Starting from April, as the sea begins to warm, the added thermal energy accelerates marine processes, leading to increased unpredictability in the SST forecasts.

**Figure 6:**
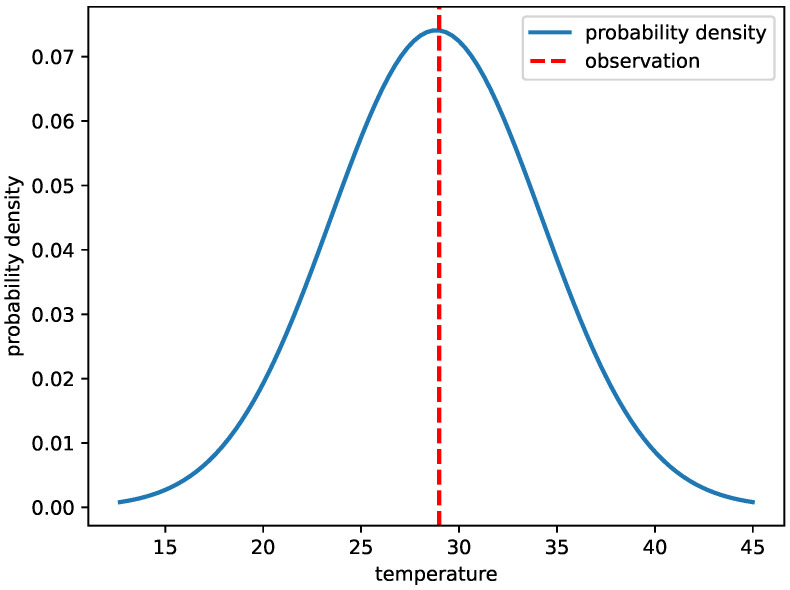
Probability density functions for the results.

Figure 6 showcases the probability density functions (PDFs) [48] derived from the LSTM-GPR model at four equidistant intervals within the Bohai Sea’s testing dataset. A striking feature of these PDFs is their uniformity. They neither exhibit pronounced peaks nor valleys and maintain consistent widths, suggesting that the probability densities generated by LSTM-GPR are well calibrated. The proximity of the observation lines to the central region of these curves further underscores the model’s high predictive accuracy for these specific instances.

**Figure 7:**
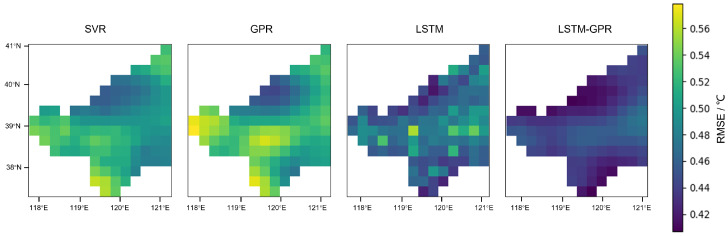
RMSE comparison for different models in the Bohai Sea region. The figure shows the spatial distribution of RMSE for four models: SVR, GPR, LSTM, and LSTM-GPR.

To further demonstrate the effectiveness of our model, we present the spatial distribution of RMSE for different models in the Bohai Sea region, as shown in Figure 7. This figure illustrates the RMSE distribution for each model, with the LSTM-GPR method consistently outperforming the other approaches. The results highlight that the proposed LSTM-GPR model effectively reduces prediction errors, even in challenging coastal areas with complex spatial characteristics. Additionally, Table 2 presents the MAE and RMSE for each method, providing further evidence of the robustness and superior performance of our model in coastal regions.

**Figure 8:**
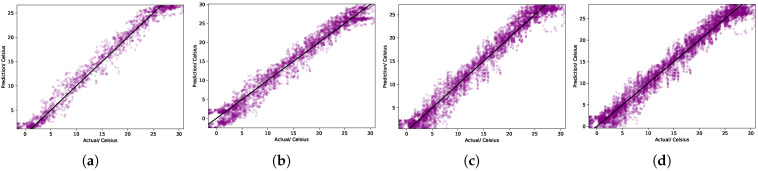
Scatter plots of predicted vs. actual SST values for each method (7-day forecast) in the South China Sea. The diagonal line indicates a perfect prediction. The RMSE and correlation coefficients ($R^{2}$) for each method are shown in (**a**) SVR (RMSE = 0.63, $R^{2}=0.79$); (**b**) GPR (RMSE = 0.65, $R^{2}=0.77$); (**c**) LSTM (RMSE = 0.70, $R^{2}=0.81$); and (**d**) LSTM-GPR (RMSE = 0.62, $R^{2}=0.80$).

In Figure 8, we present scatter plots comparing the predicted SST values to the actual observed values for a designated location within the Bohai Sea. These plots encompass predictions from SVR, GPR, LSTM, and our LSTM-GPR model. A hallmark of superior predictive performance is the close alignment of data points to the diagonal line, indicating minimal deviation between predicted and actual values. As evident in Figure 8d, the data points corresponding to the LSTM-GPR model are densely clustered around this diagonal, underscoring its enhanced predictive prowess. This superior performance aligns with our expectations, given that our model leverages GPR to further refine spatial features, building upon the foundational strengths of LSTM. This synergy results in SST predictions that are both precise and reliable.

#### 3.3.3. Other Experiments

SST is not an isolated phenomenon. It is intricately influenced by a myriad of external factors [49]. Among these, wind speed plays a pivotal role in modulating the temperature of the ocean’s surface. To delve deeper into the interplay between wind speed and SST, and to further refine the predictive capabilities of our LSTM-GPR model, we decided to incorporate wind-speed metrics, specifically USSW (east–west wind speed) and VSSW (north–south wind speed), into our predictive framework.

We modified our LSTM model to ingest these wind-speed [50] metrics alongside historical SST data. The model was then trained to directly output a forecast spanning 7 days into the future. Recognizing the spatial nuances in SST predictions, we still employed GPR to fuse and refine the spatial characteristics of the LSTM’s predictions. This step aimed to harness the spatial dependencies between different oceanic points, further enhancing the model’s accuracy.

The outcomes of this multivariate approach are tabulated in Table 3. Comparing these outcomes with our earlier univariate predictions highlights the benefits of integrating wind speed metrics into SST forecasting. This improvement can be attributed to wind speed’s role in enhancing the heat exchange at the ocean surface, improving vertical mixing, and capturing key seasonal climatic effects that are critical for accurate SST prediction.

## 4. Conclusions and Future Work

This paper proposes a novel framework for ocean temperature prediction that integrates spatiotemporal correlations. The methodology unfolds in stages: Initially, the LSTM network, trained on historical data, extracts temporal patterns and forecasts across the dataset, yielding preliminary prediction outcomes. Subsequently, the GP models the spatial dependency between each data point and its surrounding areas from the preliminary predictions to refine the SST forecasts, and estimates confidence intervals for each prediction. To evaluate the effectiveness of our method, experimental evaluations are conducted in two distinct oceanic regions. We compare our results with existing approaches as benchmarks. Our findings highlight the superior performance of the LSTM-GPR method in ocean temperature forecasting when compared to alternative techniques.

## Figures and Tables

**Table 1:** Comparison of seven-day forecast results in the Bohai Sea and South China Sea regions.

Study Area	Model Name	Metrics	Day = 1	Day = 2	Day = 3	Day = 4	Day = 5	Day = 6	Day = 7
Bohai Sea	SVR	RMSE	0.5041	0.7824	0.9587	1.0939	1.2164	1.3309	1.4439
		MAE	0.3526	0.5789	0.7261	0.8391	0.9429	1.0409	1.1397
		$R^{2}$	0.9964	0.9913	0.9869	0.9829	0.9789	0.9748	0.9703
	GPR	RMSE	0.5163	0.7859	0.9565	1.0864	1.2040	1.3146	1.4221
		MAE	0.3443	0.5586	0.6987	0.8045	0.9005	0.9907	1.0798
		$R^{2}$	0.9962	0.9911	0.9869	0.9831	0.9792	0.9753	0.9710
	LSTM	RMSE	0.4679	0.7707	0.9963	1.1914	1.3832	1.5740	1.7632
		MAE	0.3239	0.5797	0.7739	0.9402	1.1014	1.2606	1.4186
		$R^{2}$	0.9968	0.9914	0.9857	0.9794	0.9722	0.9639	0.9545
	LSTM-GPR	RMSE	**0.4384**	**0.6737**	**0.8171**	**0.9202**	**1.0117**	**1.0966**	**1.1710**
		MAE	**0.2954**	**0.4905**	**0.6105**	**0.6937**	**0.7666**	**0.8351**	**0.8967**
		$R^{2}$	**0.9972**	**0.9935**	**0.9904**	**0.9879**	**0.9853**	**0.9828**	**0.9803**
South China Sea	SVR	RMSE	0.2569	0.3902	0.4666	0.5191	0.5616	0.5993	0.6335
		MAE	0.1763	0.2876	0.3537	0.3986	0.4346	0.4660	0.4945
		$R^{2}$	0.9638	0.9183	0.8851	0.8593	0.8364	0.8142	0.7930
	GPR	RMSE	0.2740	0.4095	0.4869	0.5403	0.5836	0.6223	0.6578
		MAE	0.1848	0.2991	0.3668	0.4131	0.4502	0.4827	0.5123
		$R^{2}$	0.9574	0.9080	0.8725	0.8449	0.8207	0.7972	0.7745
	LSTM	RMSE	0.2560	0.4030	0.4948	0.5605	0.6151	0.6641	0.7094
		MAE	0.1820	0.3057	0.3838	0.4390	0.4842	0.5247	0.5621
		$R^{2}$	0.9647	0.9251	0.8948	0.8715	0.8512	0.8324	0.8149
	LSTM-GPR	RMSE	**0.2474**	**0.3792**	**0.4572**	**0.5117**	**0.5562**	**0.5950**	**0.6296**
		MAE	**0.1734**	**0.2838**	**0.3506**	**0.3965**	**0.4334**	**0.4651**	**0.4937**
		$R^{2}$	**0.9669**	**0.9234**	**0.8902**	**0.8638**	**0.8401**	**0.8179**	**0.7970**

**Entries in bold** represent the best results for each metric and forecast horizon.

**Table 2:** MAE and RMSE of different methods in Bohai coastal areas.

Method	SVR	GPR	LSTM	LSTM-GPR
MAE	0.3637	0.3497	0.3168	0.2916
RMSE	0.5093	0.5175	0.4548	0.4293

**Table 3:** Comparison of seven-day forecast results in the Bohai Sea and South China Sea regions. RMSE and MAE are presented in degrees Celsius (°C).

Study Area	Model Name	Metrics	Day = 1	Day = 2	Day = 3	Day = 4	Day = 5	Day = 6	Day = 7
Bohai Sea	LSTM-GPR (SST)	RMSE	**0.4384**	0.6737	0.8171	0.9202	1.0117	1.0966	1.1710
		MAE	**0.2954**	0.4905	0.6105	0.6937	0.7666	0.8351	0.8967
		$R^{2}$	**0.9972**	0.9935	0.9904	0.9879	0.9853	0.9828	0.9803
	LSTM-GPR (SST, USSW, VSSW)	RMSE	0.4733	**0.6595**	**0.7700**	**0.8487**	**0.9129**	**0.9669**	**1.0139**
		MAE	0.3410	**0.4885**	**0.5766**	**0.6383**	**0.6884**	**0.7321**	**0.7711**
		$R^{2}$	0.9968	**0.9937**	**0.9915**	**0.9896**	**0.9880**	**0.9865**	**0.9852**
South China Sea	LSTM-GPR (SST)	RMSE	0.2474	0.3792	0.4572	0.5117	0.5562	0.5950	0.6296
		MAE	**0.1734**	0.2838	0.3506	0.3965	0.4334	0.4651	0.4937
		$R^{2}$	0.9669	0.9234	0.8902	0.8638	0.8401	0.8179	0.7970
	LSTM-GPR (SST, USSW, VSSW)	RMSE	**0.2443**	**0.3643**	**0.4372**	**0.4879**	**0.5292**	**0.5655**	**0.5988**
		MAE	0.1773	**0.2770**	**0.3377**	**0.3794**	**0.4131**	**0.4427**	**0.4696**
		$R^{2}$	**0.9673**	**0.9282**	**0.8983**	**0.8749**	**0.8538**	**0.8334**	**0.8132**

**Entries in bold** represent the best results for each metric and forecast horizon.

## Data Availability

Dataset available on request from the authors.
